# Graduate Study on the Continent—Russia

**Published:** 1909-04-10

**Authors:** 


### GRADUATE STUDY ON THE CONTINENT—RUSSIA.

### II.—GRADUATE STUDY IN RUSSIA.

In the former article we briefly described what the graduate student may derive from a visit to Russia. In this we propose to deal with the facilities the Russian practitioner (and also the foreigner who, understanding Russian, is in a position to avail himself of these facilities) is given for the prosecution of graduate study in private practice. To some of our readers it may be a surprise to hear that in the matter of post-graduate study associations Russia is well in the front rank of European countries. As early as 1875 a movement was originated in St. Petersburg having for its object the promotion of graduate study both in medicine and in dentistry, and Russia may therefore claim to have been one of the first countries to recognise the necessity of such study, and to encourage it by giving the general practitioner opportunities for indulging in it. The first definite step towards initiating this movement was made by a special committee composed of the leaders of the medical profession in Russia, private practitioners, and lay friends who lent their support, both sympathetic and financial, to the movement. Among these lay friends the names of several members of the reigning house, and especially of the Grand Duchess Helene Pawlovna, figured prominently. Under their patronage was built and endowed what was probably the first post-graduate study institution in Europe, the fine Imperial Clinical "Helene Pawlovna Institute" at St. Petersburg. This institution, designed as a centre for post-graduate instruction, was founded in 1875, and built out of a fund of 80,000 roubles collected from private sources, to which, by command of the Czar Alexander II., a State contribution, amounting to 40,000 roubles annually, was added. In May, 1885, the Institute, erected at a total cost of 500,000 roubles, was formally opened. The original site was granted to the committee by the Ministry for War, and from private sources a sum of 150,000 roubles was contributed as the nucleus of a permanent endowment fund. Since 1885 this fine institution has gradually been enlarged and improved, the last addition, a building devoted to pathological anatomy, being completed in 1905. At present the Institute comprises seven large quadrangular buildings and three wooden pavilions, which serve as clinical wards for special cases. There are departments for pure medicine, midwifery, surgery, pathological anatomy, and bacteriology, and out-patient departments for the other specialities, which include special clinics for dermatology, syphilology, genito-urinary diseases, ophthalmology, otology, laryngology, rhinology, and psychiatry. At the head of each department is a special demonstrator, who has the rank and receives the stipend (3,000 roubles) of an ordinary University professor, and is required to give at least three free demonstrations or lectures weekly. In addition, the teaching staff comprises eight honorary or consulting professors and two demonstrators who receive no salary, but who are allowed to give private courses, for which a small fee is charged. Practitioners may attend all clinical lectures, demonstrations, and practical courses on payment of a half-yearly subscription of ten roubles; from this source the Institute derives a yearly income of 3,000 roubles. Patients attending the clinics are charged a small fee; for Röntgen-ray work done on behalf of private patients a fee is also charged. In addition the Institute receives a yearly grant from the Government, and a sum accrues annually from the interest on the permanent endowment fund, while there are usually private subscribers. The Institute is open all the year round, with the exception of a summer vacation from May 15 to September 1. Most of the practitioners, who attend from all parts of Russia, usually take a very active share in the work. Thus we are informed that in many cases the activity of the students is so great that the practical courses last till 11 o'clock at night in some instances. Courses are given in all subjects, usually according to the demand of the students, and practical courses are limited to ten members. It is intended to enlarge the Institute considerably, as the out-patient departments are usually overcrowded, both the number of patients and the number of students attending being larger than the present accomodation can cope with. An interesting fact is that 40 per cent. of the students are country practitioners who regularly come to the city to take part in these courses. In many cases even the small subscription asked is not taken, the Director having the privilege of admitting practitioners who cannot pay this amount as free guest auditors. About 4 per cent. of students take advantage of this proviso. No examinations are held or diplomas granted, but an exception to this is made in the case of municipal officers, who are granted a period of leave of absence, with full salary, to attend the courses, and who desire a certificate showing that they have spent their leave in attending the clinics. A feature of the institution is the *colloquium*, or general discussion, on some special case or subject, in which practitioners are invited to bring forward their views, criticise opinions, and generally discuss the matter by means of question and answer.

In addition to this fine and highly successful institution (for the particulars of which we are indebted to the report of the Director, Dr. Tilling, courteously placed at our disposal by Professor R. Kutner, of Berlin) there is at St. Petersburg another special institution devoted to post-graduate study. This is the

Imperial Clinical Gynæcological Institute, at which regular and periodical post-graduate courses are given. Practitioners have the privilege of working in the Institute, assisting at operations, participating in the practical courses, and attending the theoretical demonstrations and lectures. The hospital itself, situate on the Wassil-jewsky-Ostrow, is one of the oldest in the city, dating back to the eighteenth century; but the new buildings are entirely modern, and at present the Institute is unrivalled as a gynæcological hospital. The lecture room, wards, operating room, delivery ward, museum, library, electric light, *x*-ray room, hydro-therapeutic department, and the laboratories are arranged and equipped in the most modern fashion and on the best principles, and the whole has cost more than £300,000. The courses are given in Russian, but many of the professors speak German and English, and it is possible that the English graduate may find opportunities and facilities for work here. The large amount of clinical material and the excellence of the institution certainly make it one of the most interesting hospitals in Northern Europe. A large number of practitioners avail themselves of the unrivalled opportunities for post-graduate work offered here. The staff is large and the teaching excellent, and a feature of the institution is that it affords the graduate so much chance for perfecting his practical knowledge by actual practical work in midwifery.

A somewhat similar though hardly so fine or so well-equipped institution exists at Moscow. This is the

Gynæcological Institute for Practising Physicians, an institution entirely devoted to the interests of the qualified man in general practice. This fine hospital owes its existence in the first instance to the munificence of a layman, M. Schelaputin, of Moscow, who offered to build and endow a hospital in which practising physicians would have opportunities for perfecting themselves in the practice of midwifery. The offer was gladly accepted by Professor Snegireff, one of the best-known Russian gynæcologists, and in 1896 the hospital was formally opened. It accommodates close upon forty patients, and comprises sixteen small wards, three fine operating rooms and accessories. Regular courses are given here, the number of participants in each course, which is essentially practical, being, for obvious purposes, strictly limited. These courses are, of course, entirely devoted to gynæcological subjects, but they deal with all questions connected with this speciality. Thus, to take the last series of courses, the programme comprised the following :—Anatomy of the female genital organs and of the pelvis; diagnosis of abdominal and pelvic tumours; gynæcological massage; gynæcological electro-therapeutics; operative gynæcology with operations on the cadaver; the nursing of gynæcological patients; after-treatment; difficult labour; abortion; midwifery course, with practical operations; pathological course, microscopic and macroscopic. These courses are very popular, and the list is always full, far more practitioners applying than can possibly be allowed to participate.

At Warsaw, Kiev, and Odessa, and also at Helsingfors, courses are from time to time given for the benefit of practising physicians. The large amount of valuable clinical material available at the fine hospitals possessed by the first-mentioned three cities makes these courses, which are well attended, exceedingly valuable. Furthermore, the Russian practitioner is well catered for by the special courses given at German and Austrian centres, practically just over the border. Among these may be mentioned the courses given in the northern German centres, such as Rostock, Kiel, and Königsberg, and in the more southern university towns, Breslau and Cracow.

In conclusion it may be added that it is proposed to establish at Moscow a large "Institute for Social Hygiene" in which practical courses may be given with the object of instructing practitioners, and under certain conditions laymen whose work brings them into contact with the public, on all questions affecting the health of the community. The scheme has not been fully worked out, and details are therefore not yet available; but the proposal has the support of the whole profession in Russia, which, through the Pirogoff Medical Association, has initiated the movement. It is pointed out that graduate study has a decided interest for the community. The better the doctor is equipped with practical knowledge the better is he able to work for the public welfare. To a large extent hygienic conditions in the smaller Russian towns leave much to be desired, and such an institution will undoubtedly be able to ameliorate the existing condition of things.

Enough has been said to show that graduate study in Russia is by no means neglected. It is true the foreign graduate will not be attracted, at present at least, to that country, as he is towards Germany, France, or Austria, but for the local practitioner the opportunities and facilities offered by the existing institutions are so great and so excellent that the English practitioner may well envy his Russian colleague.

